# Long noncoding RNA HOTAIR promotes breast cancer development by targeting ZEB1 via sponging miR-601

**DOI:** 10.1186/s12935-020-01410-9

**Published:** 2020-07-17

**Authors:** Yuanyuan Wang, Guoliang Gong, Jingyun Xu, Yuanxin Zhang, Shenggui Wu, Shaohong Wang

**Affiliations:** 1 Department of Pathology, Shantou Central Hospital and Affiliated Shantou Hospital of Sun Yat-Sen University, Shantou, 515000 China; 2 Department of Pathology, The First Affiliated Hospital of Shantou University Medical College, No. 57, Changping Road, Shantou, 515041 Guangdong China; 3 Department of Pathology, Chaonan Minsheng Hospital of Shantou, Shantou, 515000 China

**Keywords:** BC, HOTAIR, miR-601, ZEB1, AKT signaling pathway

## Abstract

**Background:**

Breast cancer (BC) is a common malignancy worldwide. It has been reported that long non-coding RNA (lncRNA) HOX transcript antisense RNA (HOTAIR) is abnormally expressed in BC. However, the role of HOTAIR in the malignancy of BC is worth further discussion. This study aims to clarify the function and molecular mechanism of HOTAIR in BC.

**Methods:**

Quantitative real-time polymerase chain reaction (qRT-PCR) was employed to determine the expression of HOTAIR, microRNA (miR)-601 and zinc finger E-box binding homeobox 1 (ZEB1). Cell counting kit-8 (CCK-8) and transwell assay were used to detect the proliferation, migration and invasion of cells. Further, the protein levels of AKT, phosphorylated-AKT (p-AKT), ZEB1 and Ki-67 were confirmed by western blot (WB) assay. Moreover, dual-luciferase reporter assay was applied to examine the targeting relationship between HOTAIR and miR-601 or miR-601 and ZEB1. In addition, animal experiments were conducted to verify the effect of HOTAIR on BC tumor growth in vivo.

**Results:**

HOTAIR was upregulated in BC tissues and cells, and its knockdown suppressed the proliferation, migration, invasion and the activity of AKT signaling pathway of BC cells. HOTAIR could serve as a sponge of miR-601. Further experiments revealed that miR-601 inhibitor could reverse the inhibition effect of HOTAIR silencing on the progression of BC. Meanwhile, ZEB1 was a target of miR-601, and its overexpression could invert the suppression effect of miR-601 overexpression on the progression of BC. Additionally, ZEB1 expression was regulated by HOTAIR and miR-601. Furthermore, interference of HOTAIR could attenuate BC tumor growth in vivo.

**Conclusion:**

In short, this study demonstrated that HOTAIR promoted the proliferation, migration, invasion of BC through regulating the miR-601/ZEB1 axis, which provided a theoretical basis for the research on lncRNA-directed therapeutics in BC.

## Background

Breast cancer (BC) is one of the most common malignant tumors and the second leading cause of cancer death in women [1, 2]. The strongest risk factors of BC include age and genetic mutations [3]. Although the early diagnosis, surgical techniques and chemoradiotherapy of BC have been significantly improved, the incidence of BC is still rising steadily and becoming younger [4]. Therefore, it is urgent to elucidate the molecular mechanism of BC development to provide a theoretical basis for the prevention of BC.

Long non-coding RNA (lncRNA) is a class of functional RNA molecule with transcript length of > 200 nucleotides (nts) [5]. In recent years, studies have found that lncRNAs are widely involved in almost all physiological and pathological processes of the body, and are closely related to the occurrence and development of various tumors [6, 7]. HOX transcript antisense RNA (HOTAIR) exists in the HOX gene, and is the first lncRNA found with trans-acting. Recent studies have shown that HOTAIR abnormal expression is closely related to the progression of many tumors [8]. And it is reported that the expression of HOTAIR is extremely significant in BC-related lncRNAs [9]. Lai et al. suggested that abnormal expression of HOTAIR is associated with the poor prognosis of BC [10]. In addition, HOTAIR overexpression has been shown to promote the expression of phosphorylated-AKT (p-AKT) and thereby activate the AKT signaling pathway [11, 12]. However, the mechanism of HOTAIR in the progression of BC deserves further investigation.

Many studies have confirmed that lncRNAs can serve as the sponges of microRNAs (miRNAs) to regulate the expression of messenger RNA (mRNA) at the post-transcriptional level [13, 14]. And increasing evidence suggests that miRNAs can act as oncogenes or tumor suppressor genes to regulate the progression of many cancers [15]. MiR-601 has widely lower expressed in many cancers, including colorectal neoplasia and BC [16, 17]. Hu et al. suggested that miR-601 could suppress the growth, migration and invasion of BC cells by targeting PTP4A1 [17]. Zinc finger E-box binding homeobox 1 (ZEB1) is a zinc finger protein transcription factor encoded by Zfhxla gene. Some researches have indicated that ZEB1 can promote the proliferation and metastasis of BC cells [18], and participate in the regulation of lncRNA NEAT1 on BC progression [19]. In addition, Ma et al. reported that the overexpression of ZEB1/2 could promote the migration activity of BC cell line [20], and its down-regulation could inhibit the expression of p-AKT in adenocyte epithelial cells [21]. Therefore, the study of miR-601 and ZEB1 can help us better understand the mechanism of BC.

Our research aims to explore the role and molecular mechanism of HOTAIR in the BC process. The results of functional assays showed that knockdown of HOTAIR could inhibit the proliferation, migration, invasion and the activity of the AKT signaling pathway of BC cells in vitro, and reduce BC tumor growth in vivo. Through bioinformatics analysis and dual-luciferase reporter assay validation, we found that HOTAIR could target miR-601, and miR-601 could target ZEB1. Further rescue experiments confirmed that HOTAIR mediated the progression of BC by targeting miR-601 to regulate ZEB1 expression.

## Materials and methods

### Clinical samples and cell culture

A total of 35 BC patients were recruited to our study. Tumor samples and adjacent normal tissues were collected at Shantou Central Hospital and Affiliated Shantou Hospital of Sun Yat-Sen University, and immediately stored at − 80 °C until used. Our study was approved by the Ethics Committee of Shantou Central Hospital and Affiliated Shantou Hospital of Sun Yat-Sen University, and all enrolled patients signed informed consent.

BC cell lines (MCF-7 and MDA-MB-231) and human breast epithelial cell lines (MCF-10A) were all purchased from BeNa Culture Collection Biological Technology (Beijing, China). MCF-7 and MDA-MB-231 cells were cultured in Dulbecco’s Modified Eagle Medium (DMEM, Thermo Fisher Scientific, Waltham, MA, USA), and MCF-10A cells were cultured in RPMI-1640 (Thermo Fisher Scientific). All mediums were supplemented with 10% fetal bovine serum (FBS, Thermo Fisher Scientific) and 1% Penicillin/Streptomycin (Invitrogen, Carlsbad, CA, USA), and all cells were cultured in a 5% CO_2_ and 37 °C incubator.

### Cell transfection

Small interfering RNA (siRNA) against HOTAIR (si-HOTAIR#1/#2) and its control (si-NC), pcDNA and pcDNA-HOTAIR overexpression vector (HOTAIR) or pcDNA-ZEB1 overexpression vector (ZEB1), miR-601 mimic (miR-601), miR-601 inhibitor (in-miR-601) and matched negative controls (miR-NC, in-miR-NC) were purchased from GenePharma Co., Ltd. (Shanghai, China). Lentivirus harboring short hairpin RNA targeting HOTAIR (sh-HOTAIR) and negative control (sh-NC) were constructed by GeneCopoeia (Rockville, MD, USA). These oligonucleotides or plasmids were transfected into BC cells using Lipofectamine 2000 reagent (Invitrogen) referring to the manufacturer’s instructions.

### RNA isolation and qRT-PCR

Total RNA was extracted from tissues and cells using TRIzol reagent (Thermo Fisher Scientific) according to the manufacturer’s instructions. Extracted RNA was used to synthesize complementary DNA (cDNA) using a cDNA Reverse Transcription Kit (Thermo Fisher Scientific). QRT-PCR was carried out using SYBR^®^ Premix Dimer Eraser Kit (Takara, Dalian, China). TaqMan microRNA assays (Thermo Fisher Scientific) were used to measure miR-601 (NR_030332.1) level, and U6 small nuclear RNA (U6-snRNA) was used as the internal control. The relative expressions of HOTAIR (NR_047517.1) and ZEB1 (NP_110378.3) were calculated using the 2^−ΔΔCt^ method, and GAPDH was used as the internal control. The special primers for miR-601 or U6 were purchased from GeneCopoeia (Rockville, MD, USA) and primers for HOTAIR, ZEB1 and GAPDH were listed as below: HOTAIR, Forward: 5′-CAGTGGGGAACTCTGACTCG-3′, Reverse: 5′-GTGCCTGGTGCTCTCTTACC-3′; ZEB1, Forward: 5′-GCCAATAAGCAAACGATTCTG-3′, Reverse: 5′-TTTGGCTGGATCACTTTCAAG-3′; GAPDH, Forward: 5′-TCAAGGCTGAGAACGGGAAG-3′, Reverse: 5′-TGGACTCCACGACGTACTCA-3′.

### Western blot (WB) assay

Tissues and cells were lysed with RIPA lysis buffer (Beyotime, Shanghai, China). Total protein was quantified by BCA Protein Assay Kit (Beyotime) according to the manufacturer’s instructions. The same amount of protein was separated using SDS-PAGE gel, transferred onto polyvinylidene difluoride membranes (Millipore, Billerica, MA, USA) and blocked with 5% non-fat milk for 1 h at room temperature. Subsequently, the membranes were incubated with primary antibodies against p-AKT (1:750, Abcam, Cambridge, MA, USA), AKT (1:10,000, Abcam), ZEB1 (1:500, Abcam), Ki-67 (1:1,000, Abcam) or β-actin (1:5,000, Abcam) overnight at 4 °C, and then incubated with secondary antibody labeled with HRP (1:2000, Abcam) for 1 h at 37 °C. Then, the protein signaling was visualized by enhanced chemiluminescence chromogenic substrate (Beyotime) and quantitated by Image Lab software (Bio-Rad, Hercules, CA, USA).

### Cell counting kit-8 (CCK-8) assay

After transfection for 24 h, MCF-7 and MDA-MB-231 cells were inoculated in 96-well plates at the density of 1 × 10^4^ cells per well. At the indicated time (0, 24, 48 and 72 h), 10 µL CCK-8 solution (Sigma-Aldrich Co., St Louis, MO, USA) was added to each well and cultured for 4 h. The absorbance was measured with a spectrophotometer (Bio-Rad) at 450 nm to reflect the proliferation of cells.

### Transwell assay

The migration and invasion abilities of cells were measured by transwell assay (Corning, NY, USA). After transfection for 24 h, 1 × 10^5^ cells were seeded in the upper chamber with or without Matrigel (BD Biosciences, San Jose, CA, USA) to detect cell invasion and migration, respectively. The upper chamber was filled with 100 µL serum-free medium, while the lower chamber was added with 600 µL serum medium. After 24 h, the lower chamber was fixed with paraformaldehyde and stained with crystal violet. The number of migrated and invaded cells was counted under an inverted microscope.

### Dual-luciferase reporter assay

LncBase Predicted v.2 and DIANA tools were used to predict the binding sites between HOTAIR and miR-601 or miR-601 and ZEB1, respectively. Partial fragments of HOTAIR and ZEB1 3′UTR containing miR-601 binding sites or mutant binding sites were subcloned into the psiCHECK-2 luciferase vector (Promega, Madison, WI, USA) to produce the HOTAIR-WT/MUT and ZEB1 3′UTR-WT/MUT reporter vectors, respectively. MCF-7 and MDA-MB-231 cells were seeded in 24-well plates and co-transfected with 40 nM miR-601 or miR-NC and 100 ng corresponding luciferase reporter vector using Lipofectamine 2000. After transfection for 48 h, Dual-Luciferase Reporter Assay Kit (Promega) was used to determine the luciferase activities of cells. In brief, MCF-7 and MDA-MB-231 cells were washed with PBS, and cells were lysed for 30 min by lysis buffer PLB. After that, 10 µL of the cell lysate was added to a 96-well plate, and then 100 µL of Reagent II was added to each well, and the absorbance of the cells was measured at 550 nm, which was the Firefly luciferase reaction intensity (RLU1). Then, 100 µL stop reagent was added into each well, and the absorbance of cells was detected at 480 nm, which was the Renilla luciferase reaction intensity (RLU2). Finally, the luciferase activities of cells were the ratio of the two groups of data (RLU1/RLU2).

### RNA immunoprecipitation (RIP) assay

After transfection of MCF-7 and MDA-MB-231 cells with miR-601 or miR-NC for 48 h, RIP assay was performed using Magna RIP RNA-Binding Protein Immunoprecipitation Kit (Millipore). The cells were lysed with RIP buffer containing magnetic beads, which conjugated with the antibody against IgG (Millipore) or argonaute2 (Ago2, Millipore) overnight at 4 °C. After isolated the immunoprecipitated RNA, the enrichment of HOTAIR in IgG or Ago2 immunoprecipitated complex was detected by qRT-PCR.

### Mice transplanted models

The animal experiment was approved by the animal research committee of Shantou Central Hospital and Affiliated Shantou Hospital of Sun Yat-Sen University, and conducted in accordance with the guidelines of the national animal protection and ethics institute. Eighteen one-month-old BALB/c nude mice (male) were purchased from Beijing life river experimental animal technology Co., Ltd. (Beijing, China) and randomly divided into 3 experimental groups (n = 6 per/group). MCF-7 cells transiently transfected sh-HOTAIR or sh-NC and non-transfected MCF-7 cells (5 × 10^6^) were subcutaneously injected into the flanks of nude mice. Tumor width and length were monitored every week and using the formula (volume (mm^3^) = width^2^ × length/2) to calculate tumor volume. After 4 weeks, mice were euthanized, and tumor samples were taken for further molecular study.

### Immunohistochemistry (IHC) assay

The tumor tissue was sectioned in paraffin and immunostaining with Ki-67 IHC Kit (YaJi Bio, Shanghai, China) according to the manufacturer’s instructions. Finally, the expression of Ki-67 was observed under a microscope and photographed.

### Statistical analysis

All data were presented as the mean ± SD from 3 independent experiments. The comparisons between 2 groups were conducted by Student’s *t-*test and among multiple groups were analyzed via one-way analysis of variance (ANOVA). The difference was considered as a statistically significant at *P* value < 0.05.

## Results

### HOTAIR was highly expressed in BC tissues and cells

**Fig. 1:**
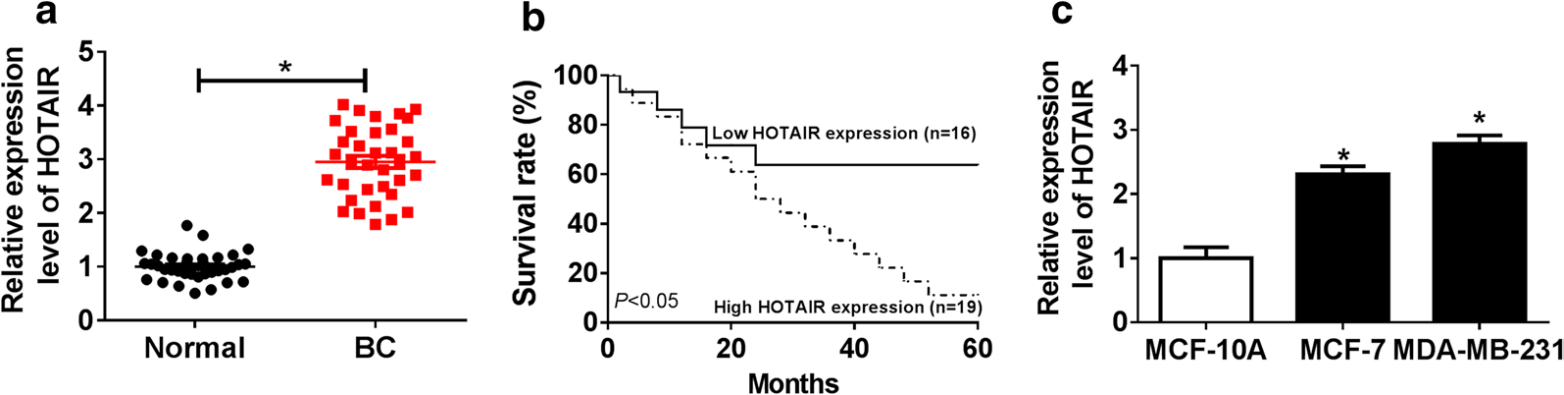
The expression of HOTAIR in BC tissues and cells. **a** The expression of HOTAIR in BC tissues and adjacent normal tissues was detected by qRT-PCR. **b** Kaplan–Meier method analysis (log-rank test) was used to analyze the correlation between HOTAIR expression level and survival rate of BC patients. **c** The expression of HOTAIR in BC cell lines (MCF-7 and MDA-MB-231) and MCF-10A cells was measured using qRT-PCR. *P < 0.05

Firstly, the expression pattern of HOTAIR was explored in BC tissues and cells. QRT-PCR assay revealed that HOTAIR level was strikingly upregulated in 35 cases of BC tissues compared with adjacent normal tissues (Fig. 1a). The correlation between HOTAIR expression and the clinicopathological characteristics of BC patients showed that high HOTAIR expression was positively correlated with the TNM stage and lymph node metastasis of BC patients (*P* < 0.05, Table 1). Additionally, Kaplan–Meier analysis indicated that compared with the low HOTAIR expression group, the high HOTAIR expression group had a lower survival rate in BC patients (Fig. 1b). Also, a notable increase of HOTAIR expression was observed in two BC cells (MCF-7 and MDA-MB-231) as compared to that in MCF-10A cells (Fig. 1c).

**Table 1:** Correlation between relative HOTAIR expression and the clinicopathological of 35 patients with breast cancer

Variable	Patients, n	HOTAIR expression		*P*-value
		Low	High	
Age, years				0.865
< 50	18	8	10	
≥ 50	17	8	9	
Menopause				0.462
Pre	16	7	9	
Post	19	9	10	
TNM stage				0.004
I–II	21	12	9	
III	14	4	10	
Lymph node metastasis				0.002
Negative	20	12	8	
Positive	15	4	11	
HER-2 status				0.432
Negative	19	9	10	
Positive	16	7	9	
ER status				0.328
Positive	20	9	11	
Negative	15	7	8	
PR status				0.239
Positive	22	10	12	
Negative	13	6	7	

### HOTAIR knockdown suppressed the proliferation, migration and invasion of BC cells

**Fig. 2:**
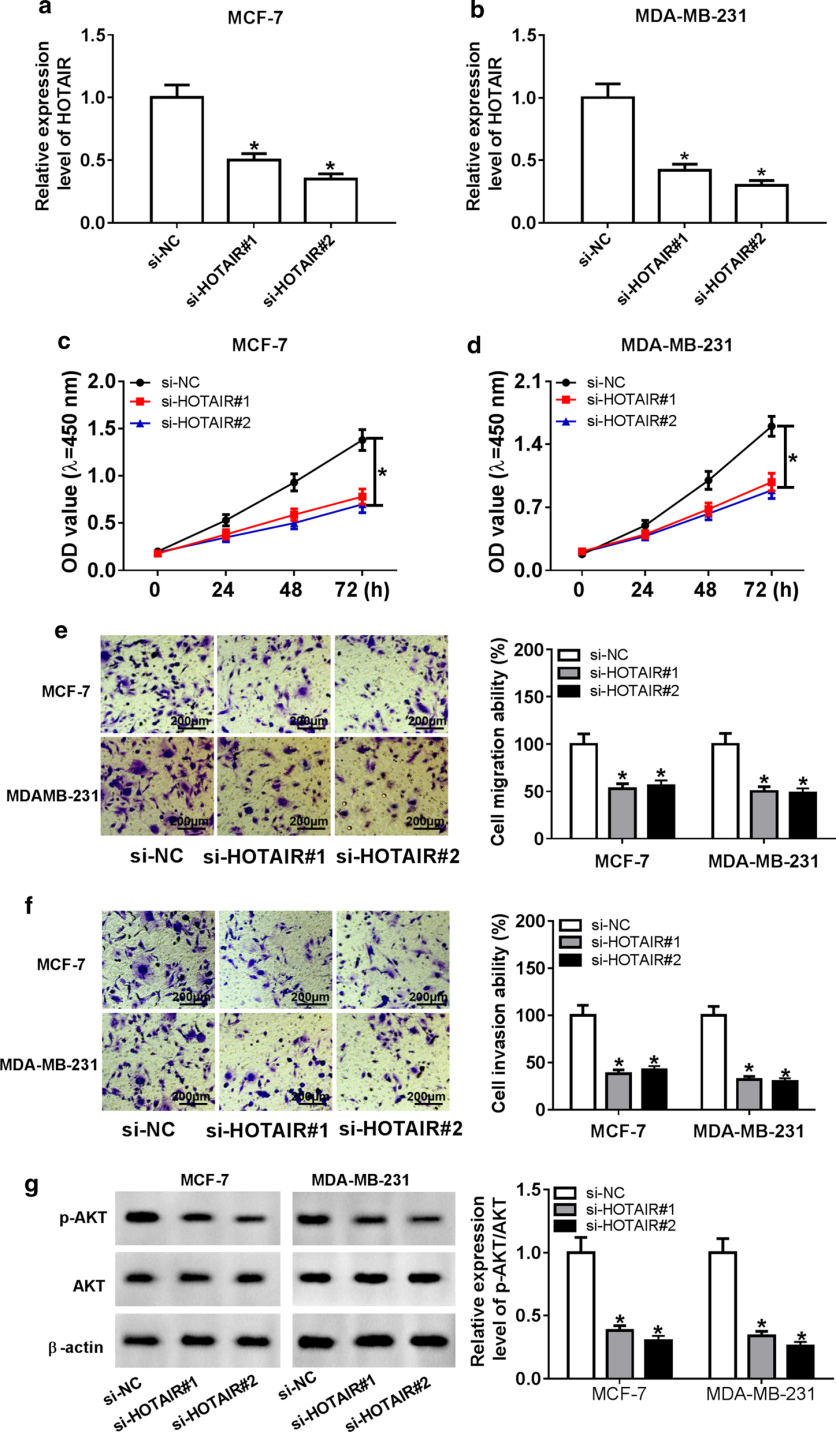
Effect of HOTAIR knockdown on BC progression. MCF-7 and MDA-MB-231 cells were transfected with si-NC, si-HOTAIR#1 or si-HOTAIR#2. **a**, **b** QRT-PCR was performed to test HOTAIR expression to assess the transfection efficiency of si-HOTAIR#1 and si-HOTAIR#2. **c**, **d** CCK-8 assay was conducted to assess the effect of HOTAIR knockdown on the proliferation ability of BC cells. **e**, **f** Transwell assay was employed to verify the suppression effect of silenced HOTAIR on the migration and invasion of BC cells. **g** The relative expression level of p-AKT/AKT was detected by WB analysis. *P < 0.05

Subsequently, we constructed siRNA to further explore the role of HOTAIR in BC. The results of qRT-PCR assay validated that the transfection of si-HOTAIR resulted in the significant reduction of HOTAIR level in MCF-7 and MDA-MB-231 cells (Fig. 2a, b), meaning that si-HOTAIR had a good transfection efficiency and could be used for the subsequent loss-of-function experiments. Then, CCK-8 assay showed that HOTAIR silencing could markedly suppress the proliferation of MCF-7 and MDA-MB-231 cells (Fig. 2c, d). Furthermore, transwell assay revealed that knockdown of HOTAIR strikingly decreased the migration and invasion abilities of MCF-7 and MDA-MB-231 cells (Fig. 2e, f). In addition, through measuring the relative expression of p-AKT/AKT, we found that silenced HOTAIR remarkably suppressed the relative expression of p-AKT/AKT, indicating that HOTAIR knockdown could restrain the activity of the AKT signaling pathway in MCF-7 and MDA-MB-231 cells (Fig. 2g).

### HOTAIR directly interacted with miR-601 in BC

**Fig. 3:**
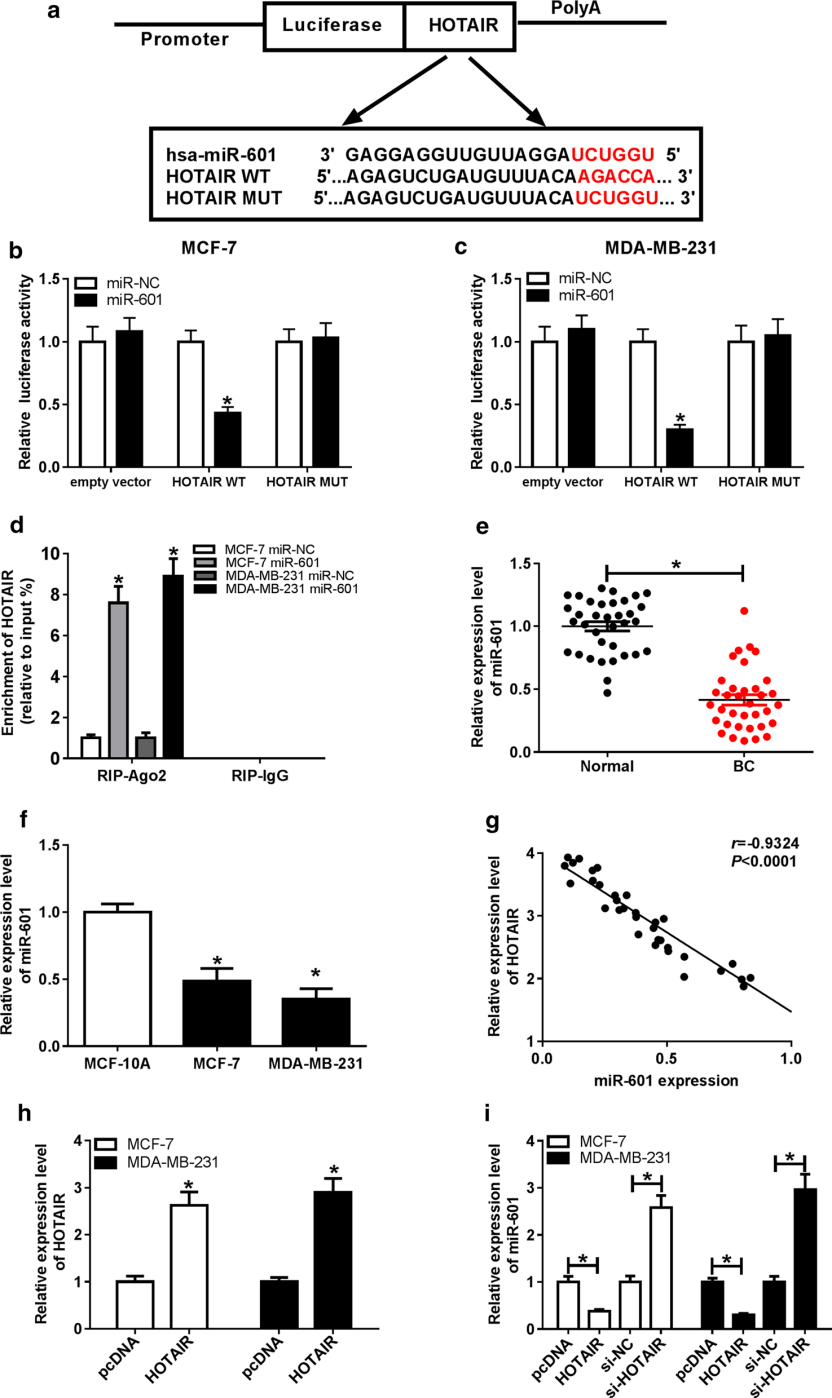
HOTAIR directly interacted with miR-601 in BC. **a** The binding sites between HOTAIR-WT/MUT and miR-601 were obtained. **b**, **c** Dual-luciferase reporter assay was carried out to test the luciferase activity of HOTAIR-WT/MUT after cells were transfected with miR-601 mimic or miR-NC. **d** RIP assay results indicated that the enrichment of HOTAIR was increased in RIP-Ago2 of BC cells transfected with miR-601. **e**, **f** MiR-601 expression was remarkably downregulated in BC tissues and cells compared to adjacent normal tissues and MCF-10A cells, respectively. **g** The negative relation between HOTAIR and miR-601 was analyzed by Pearson correlation analysis. **h** The HOTAIR overexpression efficiency was detected by qRT-PCR. **i** The expression of miR-601 was measured by qRT-PCR to evaluate the effect of HOTAIR overexpression and knockdown on miR-601 expression. *P < 0.05

In order to explore the miRNAs associated with HOTAIR, we conducted bioinformatics prediction using the LncBase Predicted v.2 tool, and found that HOTAIR contained binding sites for miR-601 (Fig. 3a). To further confirm this, dual-luciferase reporter assay was performed and the results showed that the transfection of miR-601 mimic resulted in the conspicuous downregulation of the luciferase activity of HOTAIR-WT reporter, but had no influence on the luciferase activity of HOTAIR-MUT reporter (Fig. 3b, c). Moreover, RIP assay results suggested that the introduction of miR-601 mimic resulted in the substantial enrichment of HOTAIR in RIP-Ago2 compared to RIP-IgG (Fig. 3d). Subsequently, we detected the expression of miR-601 in BC tissues and cells, and the results showed that the expression of miR-601 was significantly reduced in BC tissues and cells compared with normal tissues and cells, respectively (Fig. 3e, f). Also, correlation analysis revealed that HOTAIR expression was negatively correlated with miR-601 in BC tissues (Fig. 3g). In addition, we also measured the effect of HOTAIR expression on miR-601 expression. The elevated HOTAIR expression confirmed that the transfection efficiency of HOTAIR overexpression plasmid was good (Fig. 3h). Through detecting miR-601 expression, we uncovered that overexpression of HOTAIR significantly decreased the expression of miR-601, while knockdown of HOTAIR had an opposite effect (Fig. 3i). Taken together, these results indicated that HOTAIR directly targeted miR-601 in BC.

### The depletion of miR-601 inverted the suppression effect of HOTAIR knockdown on BC progression

**Fig. 4:**
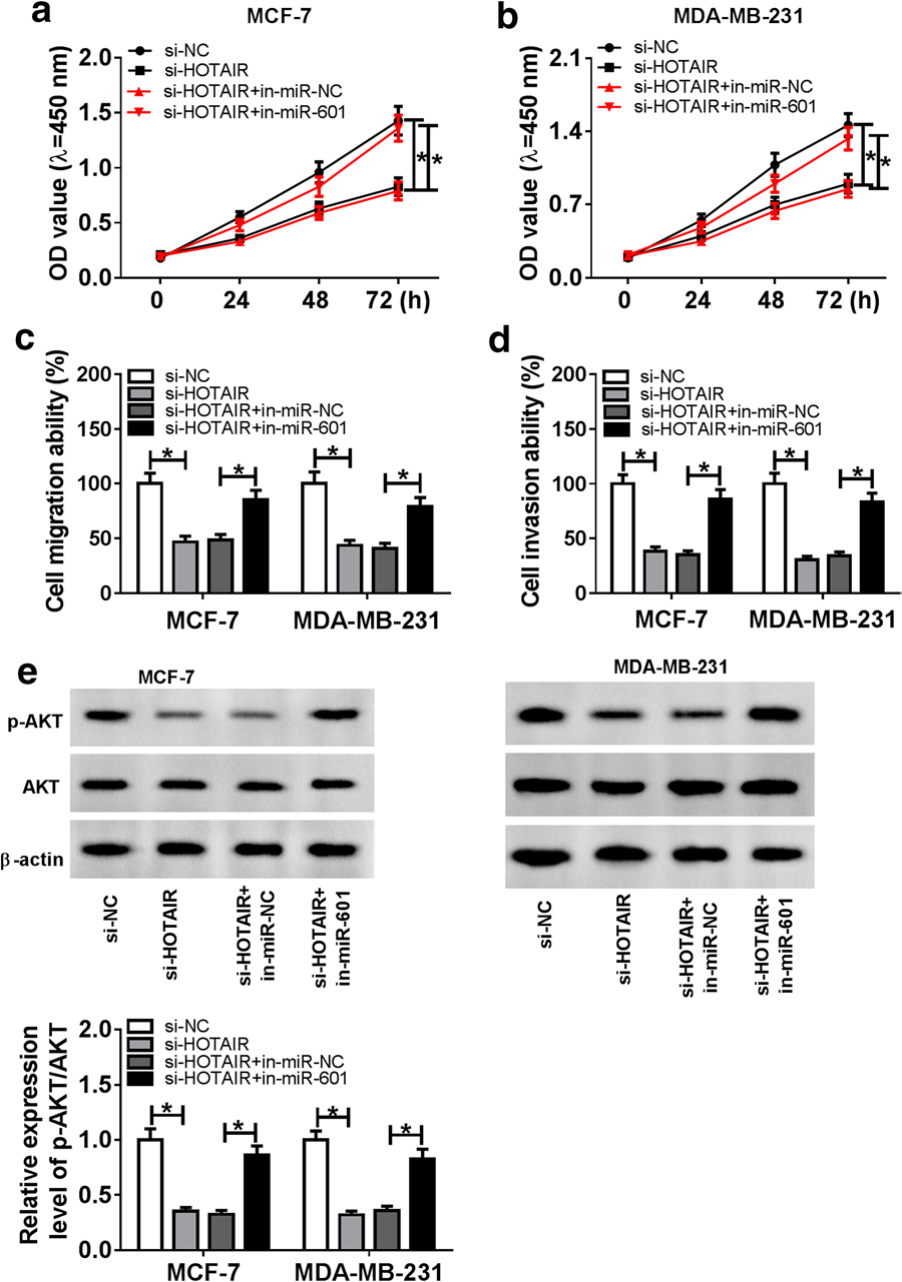
Effects of HOTAIR knockdown and miR-601 inhibitor on BC progression. MCF-7 and MDA-MB-231 cells were transfected si-NC, si-HOTAIR, si-HOTAIR + in-miR-NC and si-HOTAIR + in-miR-601, respectively. **a**, **b** CCK-8 assay was employed to evaluate the proliferation ability of BC cells. **c**, **d** Transwell assay was applied to detect the migration and invasion of BC cells. **e** Relative expression of p-AKT/AKT was tested with WB analysis. *P < 0.05

To verify whether miR-601 participated in the regulation of HOTAIR on BC progression, we co-transfected with si-HOTAIR and in-miR-601 into MCF-7 and MDA-MB-231 cells. CCK-8 assay evinced that the introduction of miR-601 inhibitor remarkably reversed the inhibition effect of HOTAIR knockdown on the proliferation ability of MCF-7 and MDA-MB-231 cells (Fig. 4a, b). Further, the suppression effect of HOTAIR silencing on the migration and invasion abilities of MCF-7 and MDA-MB-231 cells also could be inverted by miR-601 inhibitor (Fig. 4c, d). Additionally, the expression of p-AKT/AKT showed that miR-601 inhibitor also could recover the inhibition effect of HOTAIR knockdown on the AKT signaling pathway in MCF-7 and MDA-MB-231 cells (Fig. 4e). In conclusion, these data suggested that miR-601 was involved in the regulation of HOTAIR on BC progression.

### ZEB1 was a target of miR-601 in BC

**Fig. 5:**
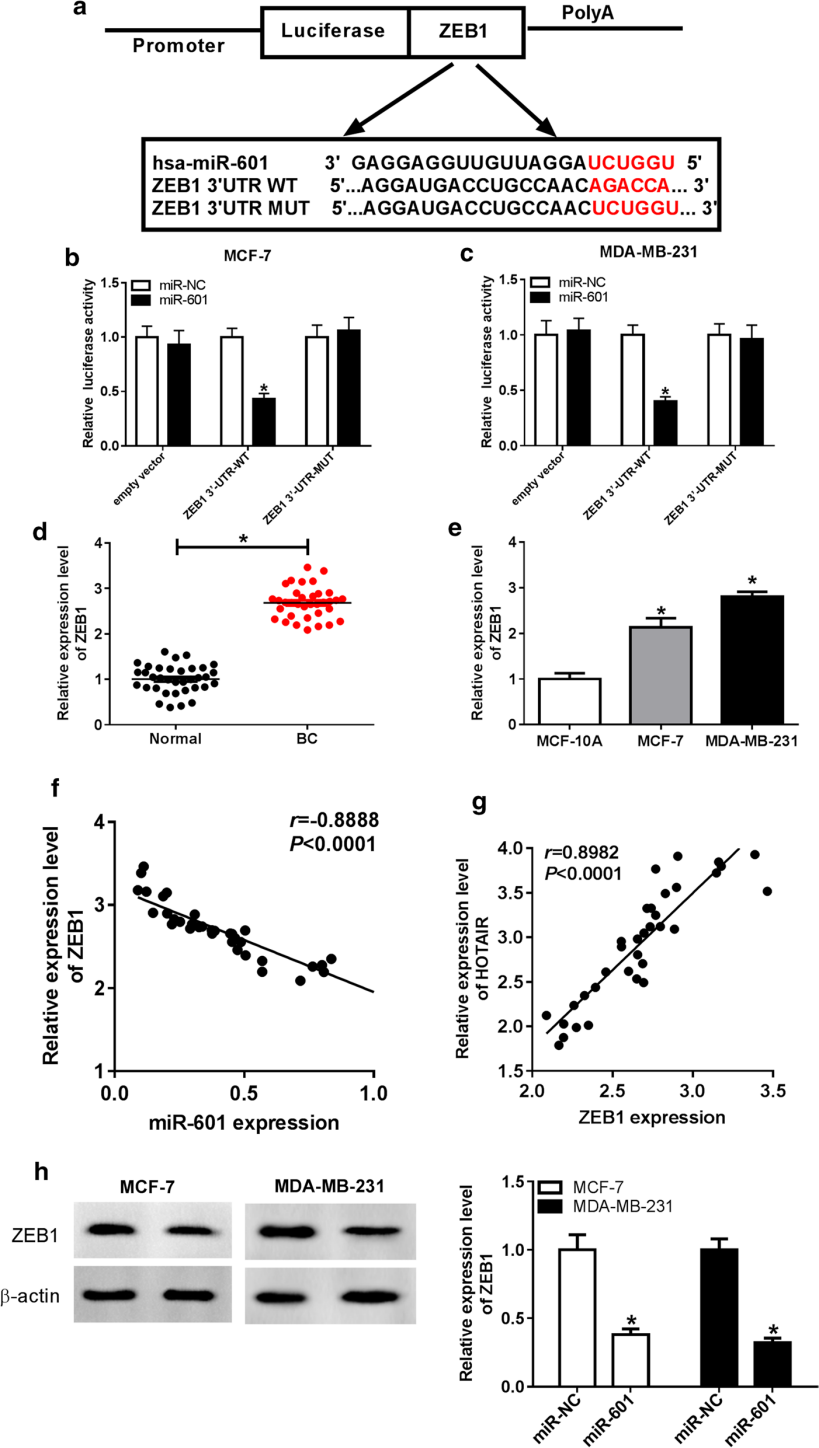
ZEB1 was a target of miR-601 in BC. **a** The binding sites between miR-601 and ZEB1 3′UTR were shown. **b**, **c** Dual-luciferase reporter assay was used to confirm the interaction between miR-601 and ZEB1 3′UTR. **d, e** ZEB1 expression was markedly upregulated in BC tissues and cells compared to adjacent normal tissues and MCF-10A cells, respectively. **f, g** Pearson correlation analysis was performed to analyze the correlation between ZEB1 and miR-601 or HOTAIR. **h** WB analysis was used to detect ZEB1 protein expression to assess the effect of miR-601 overexpression on ZEB1 expression. *P < 0.05

On the other hand, the DIANA tool was used to predict the target genes for miR-601, and found that there had existed the complementary sequences between miR-601 and ZEB1 3′UTR (Fig. 5a). Dual-luciferase reporter assay results showed that miR-601 overexpression markedly suppressed the luciferase activity of ZEB1 3′UTR-WT reporter, while had no effect on the luciferase activity of ZEB1 3′UTR-MUT reporter in MCF-7 and MDA-MB-231 cells (Fig. 5b, c). QRT-PCR assay further revealed that ZEB1 expression was strikingly upregulated in BC tissues compared with adjacent normal tissues (Fig. 5d), and its expression in MCF-7 and MDA-MB-231 cells was significantly higher than that in MCF-10A cells (Fig. 5e). Additionally, correlation analysis revealed that ZEB1 expression was reversely associated with miR-601, while positively correlated with HOTAIR in BC tissues (Fig. 5f, g). Moreover, we also found that ZEB1 expression was remarkably reduced by miR-601 overexpression in MCF-7 and MDA-MB-231 cells (Fig. 5h). These data disclosed that miR-601 could target ZEB1 in BC.

### Overexpressed ZEB1 reversed the inhibition effect of miR-601 overexpression on the proliferation, migration and invasion of BC cells

**Fig. 6:**
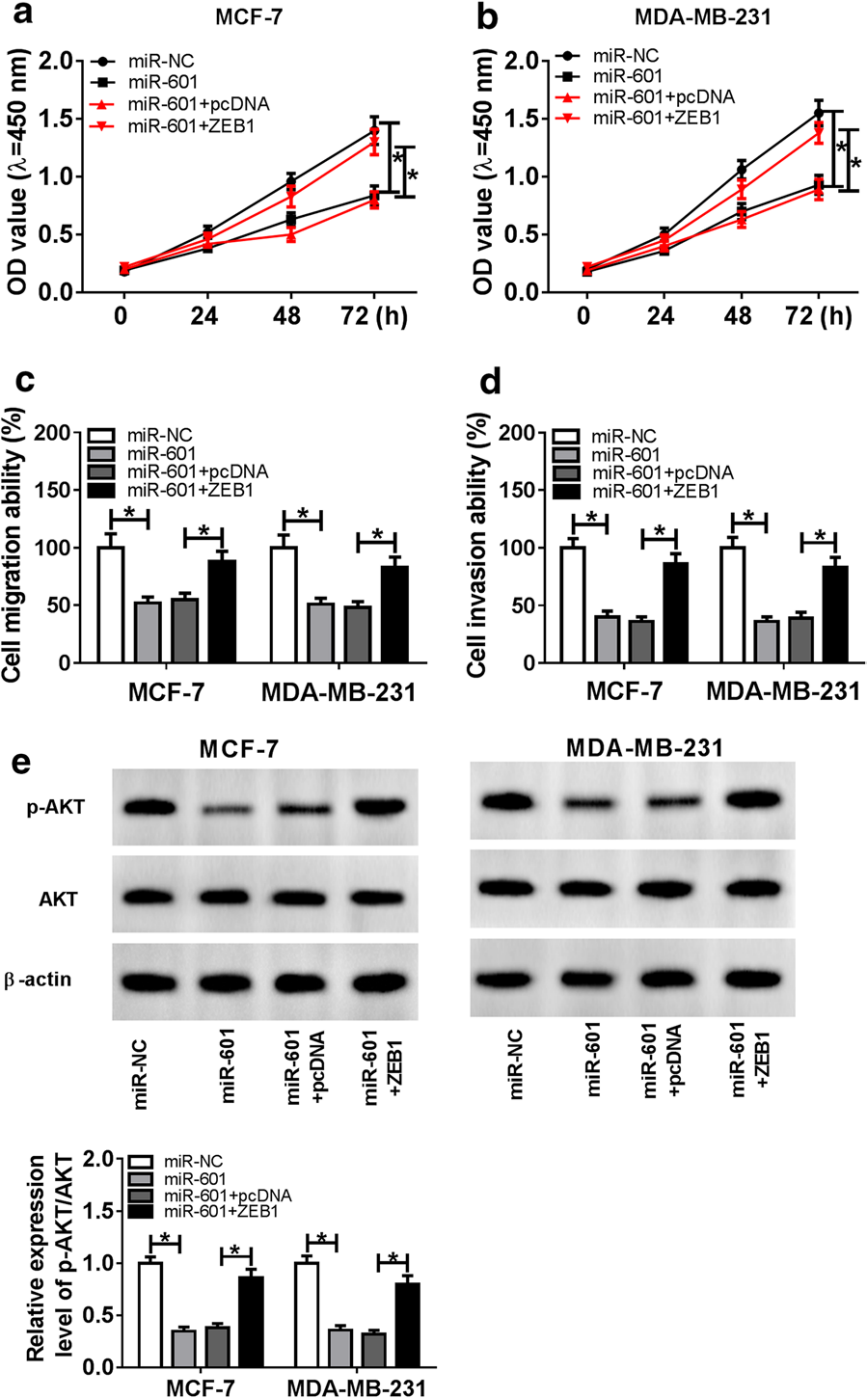
Effects of miR-601 mimic and ZEB1 overexpression on BC progression. MCF-7 and MDA-MB-231 cells were transfected with miR-NC, miR-601, miR-601 + pcDNA, miR-601 + ZEB1, respectively. **a**, **b** CCK-8 assay was performed to evaluate the proliferation ability of BC cells. **c**, **d** Transwell assay was employed to detect the migration and invasion of BC cells. **e** Relative expression of p-AKT/AKT was tested by WB analysis. *P < 0.05

To further verify whether miR-601 affected the proliferation, migration and invasion of BC cells by regulating ZEB1, we co-transfected miR-601 mimic and ZEB1 overexpression plasmid into MCF-7 and MDA-MB-231 cells. CCK-8 assay results showed that miR-601 overexpression significantly inhibited the proliferation of MCF-7 and MDA-MB-231 cells, while this effect could be inverted by the addition of ZEB1 (Fig. 6a, b). Also, ZEB1 overexpression could reverse the suppression effect of miR-601 overexpression on the migration and invasion abilities of BC cells (Fig. 6c, d). Moreover, the detection of the p-AKT/AKT protein expression showed that overexpressed ZEB1 also restored the inhibition effect of miR-601 overexpression on the AKT signaling pathway (Fig. 6e). Therefore, all data suggested that ZEB1 could participate in the progression of BC regulated by miR-601.

### ZEB1 expression was regulated by HOTAIR and miR-601

**Fig. 7:**
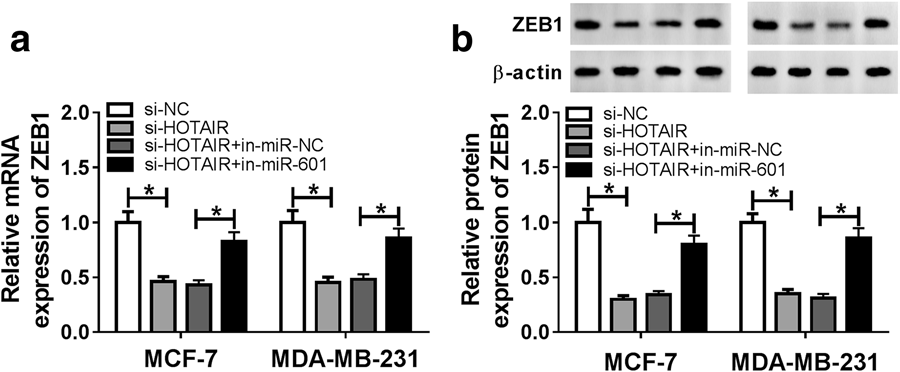
ZEB1 expression was regulated by HOTAIR and miR-601. MCF-7 and MDA-MB-231 cells were transfected si-NC, si-HOTAIR, si-HOTAIR + in-miR-NC and si-HOTAIR + in-miR-601, respectively. **a** The mRNA expression of ZEB1 was examined by qRT-PCR. **b** The protein level of ZEB1 was measured by WB analysis. *P < 0.05

To further confirm the regulation of HOTAIR on ZEB1, we measured ZEB1 expression in the presence of HOTAIR knockdown. QRT-PCR results indicated that HOTAIR knockdown could restrain the mRNA expression of ZEB1 in MCF-7 and MDA-MB-231 cells, while the addition of miR-601 inhibitor could reverse this effect (Fig. 7a). Besides, the result of ZEB1 protein level was consistent with the mRNA level (Fig. 7b). Hence, our results revealed that HOTAIR regulated ZEB1 expression by targeting miR-601.

### HOTAIR interference attenuated BC tumor growth in vivo

**Fig. 8:**
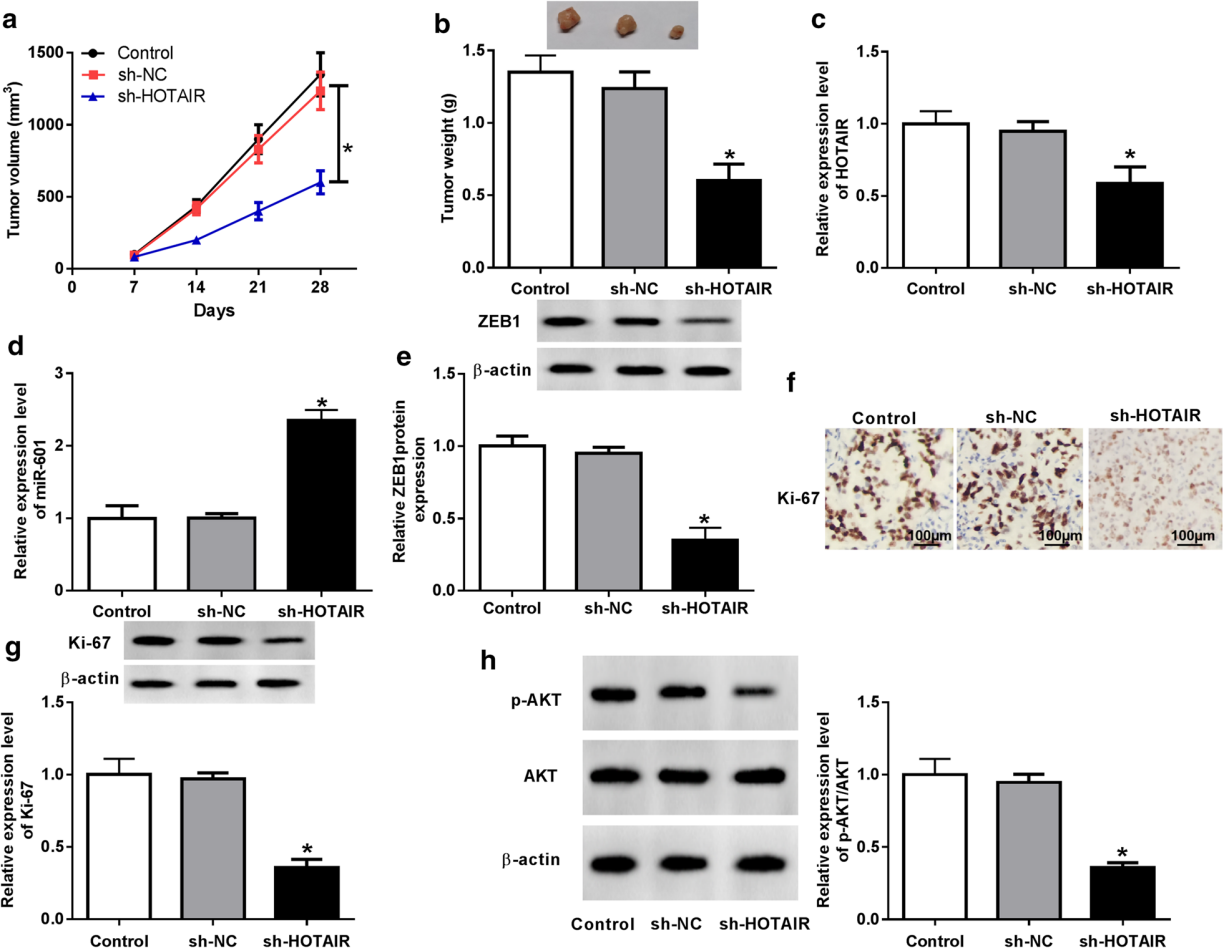
HOTAIR interference attenuated BC tumor growth in vivo. **a** Tumor volume of the sh-HOTAIR group was smaller than that of the control or sh-NC group. **b** Tumor weight was remarkably reduced in the sh-HOTAIR group compared with the control and sh-NC group. **c** The HOTAIR expression in tumor tissues was detected by qRT-PCR. **d** QRT-PCR analysis revealed that miR-601 expression in the tumor tissues of the sh-HOTAIR group was markedly higher than that in the control or sh-NC group. **e** WB assay revealed that ZEB1 protein expression in the tumor tissues of the sh-HOTAIR group was significantly lower than that in the control and sh-NC group. **f** IHC assay results revealed that Ki-67 expression was decreased in the sh-HOTAIR group. **g**, **h** The protein levels of Ki-67 and p-AKT/AKT were tested using WB analysis. *P < 0.05

To further analyze the impact of HOTAIR on BC tumor, mice transplanted models were established. By detecting the tumor volume and weight, we found that the tumor volume and weight in the sh-HOTAIR group were significantly smaller than those in the sh-NC group (Fig. 8a, b). Then, the reduction of HOTAIR expression in the sh-HOTAIR group confirmed the success of our transfection (Fig. 8c). Additionally, we also found that miR-601 expression was remarkably increased (Fig. 8d), while ZEB1 protein level was markedly decreased in the sh-HOTAIR group compared with the sh-NC group (Fig. 8e). To further determine the inhibitory effect of sh-HOTAIR on BC tumor growth, we examined the expression of proliferative marker Ki-67. IHC results revealed that Ki-67 was markedly decreased in the sh-HOTAIR group (Fig. 8f), and WB analysis results uncovered that the protein level of Ki-67 was obviously restrained in the sh-HOTAIR group (Fig. 8g). Meanwhile, the detection of p-AKT/AKT expression also indicated that the activity of the AKT signaling pathway was obviously repressed in the sh-HOTAIR group (Fig. 8f). These results revealed that HOTAIR could inhibit BC tumor growth in vivo.

## Discussion

As we all know, BC is the most common malignant tumor in females [22]. With the rise of studies on the functional properties of lncRNAs, it has been verified that lncRNAs can efficiently affect the biological processes of cancers, some of which may serve as tumor or metastasis inhibitors, such as GAS5 [23] and MEG3 [24], while others may promote oncogenesis, such as SChLAP1 [25]. LncRNA HOTAIR has been shown to interact with the polycomb repressive complex 2 (PRC2) to reprogram chromatin state and induce cancer metastasis [26, 27]. In vivo experiments show that HOTAIR can promote the invasion of breast carcinoma cells [26]. Consistent with the above research, our study found that the expression of HOTAIR was elevated in BC tissues and cells. Loss-functional experiments revealed that HOTAIR knockdown could restrain the proliferation, migration and invasion of BC cells in vitro, and reduce the tumor growth of BC in vivo. AKT signaling pathway is a classical signaling pathway associated with tumorigenesis [11]. The detection of p-AKT/AKT expression indicated that the knockdown of HOTAIR inhibited the activity of AKT signaling pathway in vitro and in vivo. Therefore, our research suggested that HOTAIR might function as a tumor promoter in the progression of BC.

In terms of mechanism, lncRNA can participate in the regulation of target genes as a competitive endogenous RNA (ceRNA) of miRNA has been recognized by researchers. Existing research showed that lncRNA HOTAIR sensitized BC cells to ionizing radiation through activating miR-218 [28]. Zhao et al. reported that HOTAIR influenced the growth, migration, invasion, and apoptosis of BC cells via the miR-20a-5p/HMGA2 axis [29]. In our study, bioinformatics predicted that HOTAIR had a binding site with miR-601, and miR-601 expression was regulated by HOTAIR in vitro and in vivo. Further experimental verification indicated that miR-601 functioned as a tumor suppressor in BC, which was consistent with previous studies on the role of miR-601 in hepatocellular carcinoma [30]. Meanwhile, inhibition of miR-601 could reverse the effect of HOTAIR knockdown on anti-proliferation, anti-migration and anti-invasion of BC cells, which was similar to the results of Hu et al. [17]. Furthermore, miR-601 inhibitor could promote the protein expression of p-AKT/AKT, indicating that miR-601 inhibitor could activate the AKT signaling pathway, which was also consistent with the study of Song et al. [30]. Hence, our research demonstrated that HOTAIR regulated the progression of BC by sponging miR-601. The anti-cancer function of miR-601 also helps us better understand the tumor-promoting effect of HOTAIR in BC.

On the other hand, we also discovered that ZEB1 was a target of miR-601. Katsura et al. showed that the downregulation of ZEB1 led to the downregulation of inflammatory cytokines and associated with the poor prognosis in BC [31]. Ma et al. showed that miR-409-3p regulated the progress of BC by targeting ZEB1 [32]. Therefore, ZEB1 was considered to be a cancer-promoting factor in many cancers. In our study, we confirmed that ZEB1 could be targeted by miR-601, and its expression was positively related to HOTAIR. The reversal effect of ZEB1 overexpression on miR-601 mimic also confirmed that it was involved in the regulation of miR-601 on BC progression. Meanwhile, we also verified that ZEB1 expression was regulated by HOTAIR and miR-601, which confirmed the existence of HOTAIR/miR-601/ZEB1 axis in BC. This is a new mechanism by which HOTAIR regulates the development of BC.

## Conclusion

In summary, our data showed that HOTAIR regulated ZEB1 expression to promote the proliferation, migration and invasion of BC cells through targeting miR-601, which mainly revealed the oncogenic function of lncRNA HOTAIR in BC. More importantly, our research enriched the lncRNA-miRNA-mRNA functional network in BC, and provided a potential therapeutic target for the treatment of BC.

## Data Availability

All data generated or analyzed during this study are included in this published article.

## Abbreviations

BC: Breast cancer
lncRNA: Long non-coding RNA
HOTAIR: HOX transcript antisense RNA
qRT-PCR: Quantitative real-time polymerase chain reaction
ZEB1: Zinc finger E-box binding homeobox 1
p-AKT: Phosphorylated-AKT
